# Content validity and methodological considerations in ecological momentary assessment studies on physical activity and sedentary behaviour: a systematic review

**DOI:** 10.1186/s12966-020-00932-9

**Published:** 2020-03-10

**Authors:** L. Degroote, A. DeSmet, I. De Bourdeaudhuij, D. Van Dyck, G. Crombez

**Affiliations:** 1 Department of Movement and Sport Sciences, Ghent University, Ghent, Belgium; 2 Research Foundation Flanders, Brussels, Belgium; 3 Department of Clinical-Experimental Health Psychology, Ghent University, Ghent, Belgium; 4 Faculty of Psychology and Educational Sciences, Free University of Brussels, Brussels, Belgium

**Keywords:** Ecological momentary assessment, Content validity, Methodological considerations, Physical activity, Sedentary behaviour

## Abstract

**Background:**

Ecological momentary assessment (EMA) is a method of collecting real-time data based on repeated measures and observations that take place in participant’s daily environment. EMA has many advantages over more traditional, retrospective questionnaires. However, EMA faces some challenges to reach its full potential. The aims of this systematic review are to (1) investigate whether and how content validity of the items (i.e. the specific questions that are part of a larger EMA questionnaire) used in EMA studies on physical activity and sedentary behaviour was assessed, and (2) provide an overview of important methodological considerations of EMA in measuring physical activity and sedentary behaviour.

**Methods:**

Thirty papers (twenty unique studies) were systematically reviewed and variables were coded and analysed within the following 4 domains: (1) Content validity, (2) Sampling approach, (3) Data input modalities and (4) Degree of EMA completion.

**Results:**

Only about half of the studies reported the specific items (*n* = 12) and the source of the items (*n* = 11). None of the studies specifically assessed the content validity of the items used. Only a minority (*n* = 5) of the studies reported any training, and one tested the comprehensibility of the EMA items. A wide variability was found in the design and methodology of the EMA. A minority of the studies (*n* = 7) reported a rationale for the used prompt frequency, time selection, and monitoring period. Retrospective assessment periods varied from ‘now’ to ‘in the last 3.5 hours’. In some studies there was a possibility to delay (*n* = 6) or deactivate (*n* = 10) the prompt, and some provided reminders after the first prompt (*n* = 9).

**Conclusions:**

Almost no EMA studies reported the content validation of the items used. We recommend using the COSMIN checklist (COnsensus-based Standards for the selection of health Measurement INstruments) to report on the content validity of EMA items. Furthermore, as often no rationale was provided for several methodological decisions, the following three recommendations are made. First, provide a rationale for choosing the sampling modalities. Second, to ensure assessment ‘in the moment’, think carefully about the retrospective assessment period, reminders, and deactivation of the prompt. Third, as high completion rates are important for representativeness of the data and generalizability of the findings, report completion rates.

**Trial registration:**

This review is registered in PROSPERO, the International prospective register of systematic reviews (registration number: CRD42017077996).

## Background

Regularly engaging in physical activity and minimizing sedentary behaviour is important in preventing and treating non-communicable diseases (NCDs), such as heart disease [1, 2], stroke [3], type II diabetes [1, 2] and breast and colon cancer [3, 4]. Notwithstanding, many adults do not meet health recommendations for physical activity and sedentary behaviour. For example, The Lancet Global Health (2018) concluded that worldwide more than one in four adults (28% or 1.4 billion people) are physically inactive [5]. Likewise, in the Western World, adults spend a large part of their day sedentary, often more than 7.5 h per day [6]. Consequently, lifestyle interventions are required that effectively promote physical activity and discourage sedentary behaviour in a large number of people at a low cost [7].

Many theoretical models posit that behaviours are the result of an interaction between individual and contextual factors [8–10]. Therefore, behaviours and their underlying determinants are best studied in their context. A detailed analysis of the psychological, social and physical environmental determinants in context may provide a profound understanding about why, how, and when these behaviours are elicited [11–13]. This is, however, not easy to accomplish. These behaviours and their accompanying determinants vary over contexts and over time, both between and within days [14–16]. Time-specific and context-specific research is therefore warranted. However, many studies use self-report questionnaires, in which participants reflect on their level of activity and its determinants, typically over an extended time period (last day/week) [17]. These self-report methods are prone to biases [18, 19]. First, participants may not accurately remember previous events or experiences, or omit details (“recall bias”) [20]. Second, questionnaires usually require that participants aggregate and summarize their responses across specific events. Consequently, variation in behaviours and determinants over time and context may be overlooked.

Ecological Momentary Assessment (EMA), also known as Experience Sampling Method (ESM), has become popular to measure physical activity and sedentary behaviours and their determinants. It involves the measurement of behaviours and experiences in naturalistic settings [21]. During EMA, users are repeatedly prompted to report on their experience and/or behaviour at fixed and/or random times per day (time-sampling), or the prompt is being triggered by a specific event (event-sampling) [22]. EMA is less susceptible to recall bias because of a lower reliance on the memory of the participants [23]. Furthermore, it may provide insights in time-varying dynamics of behaviour and its correlates [24, 25]; and provide more ecologically relevant data [26].

Despite its potential, EMA faces some challenges. First, the validity of the items used to measure certain constructs needs careful consideration. Important forms of validity to consider in EMA are construct and content validity. Construct validity is the degree to which an instrument relates with measures of the construct it claims, or purports, to be measuring [27]. Content validity is the degree to which the measure represents all facets of a construct and captures the construct in its entirety [28]. Content validity is an often neglected, but quintessential form of validity. A main premise for validity of any type of measurement is that the content of the used items reflects the construct that they are aiming to measure. Therefore, the content validity of instruments should be carefully considered before making sense of data and demonstrating other forms of validity [29]. In this systematic review, we only focus on content validity of EMA as this is a main premise for all other forms of validity, including construct validity.

As EMA is characterized by short, often repeated assessments in daily life, the items need to be considered carefully. Items from traditional questionnaires cannot simply be selected as they are often not suited for these short, repeated assessments in daily life, and therefore not by default valid for use in EMA. Assessing content validity of the items specifically used for EMA is essential when developing an EMA. In addition, EMA protocols are complex, and many decisions in terms of design and methodology need to be made (e.g. sampling type, prompt frequency, monitoring period, device). Non-compliance is a potential threat for EMA methodologies [30, 31]. Therefore, well-considered design choices have to be made to achieve sufficient data richness for the questions under study without over-burdening the participants [23, 32].

In this systematic review, we focus on the above mentioned challenges in EMA research on physical activity and sedentary behaviour in adults, for three reasons. First, the number of studies will be more feasible when only including EMA studies assessing physical activity, sedentary behaviour and their determinants. Second, this topic is highly relevant, as the literature shows an increased use of EMA within the domain of physical activity and sedentary behaviour. Third, due to the momentary assessment, recall biases are less likely, which will result in less over- or underreporting compared to self-reported measures of physical activity and sedentary behaviour.

This systematic review has two aims. First, we wanted to investigate whether and how the content validity of the items used in EMA studies on physical activity and sedentary behaviour was assessed. Second, we wanted to provide an overview of the design features of EMA in three domains, i.e. sampling approach, data input modalities and EMA completion. This review does not aim to provide conclusive answers regarding the most effective EMA methodology. Transparent and exhaustive methodological reporting can help to advance the quality and validity of this research domain and ensure that EMA methodologies reach their full potential [33].

## Methods

### Search strategy

Articles were searched in the following online databases: Pubmed, Web of Science, CINAHL, SportDiscus using the following search terms: (Physical*-activ* OR Physical-exercise OR exercise OR MVPA OR moderate-to-vigorous-physical-activity) OR (Sedentar* OR Sitting OR Physical*-inactiv* OR Screen-time OR Screentime OR Television OR TV OR Video-game OR Video-games OR Videogame* OR Gaming OR Computer-use OR Computer-time) AND (EMA OR Ecological-Momentary-Assessment OR Diary-Study OR Diaries OR Ambulatory-assessment OR Ambulatory-Monitoring OR ESM OR Experience-Sampling-Method OR Electronic-Diary OR Computer-Assisted-Diary OR Electronic-Momentary-Assessment). The search was conducted in June 2018; no other restrictions were placed on publication date. Additionally, references of literature reviews and meta-analyses on this topic were hand-searched to complement the search results. This review is registered in PROSPERO, the International prospective register of systematic reviews (registration number: CRD42017077996).

### Inclusion criteria

To be included, studies had to meet the following inclusion criteria: (1) to use EMA to assess physical activity or/and sedentary behaviour and/or their psychosocial and environmental determinants; (2) to use an electronic device as platform for the EMA; excluding studies using pencil and paper; (3) to comprise healthy people; excluding studies in clinical samples, e.g. people with insomnia, posttraumatic stress disorder, schizophrenia, etc. as this review focused on health promotion. To only include healthy people, the sample characteristics, described in the papers, were consulted; (4) to target populations of any age; (5) to have an observational, interventional, validity or feasibility study design; excluding reviews of the literature and meta-analyses; and (6) to be published in English.

### Study selection

**Fig. 1:**
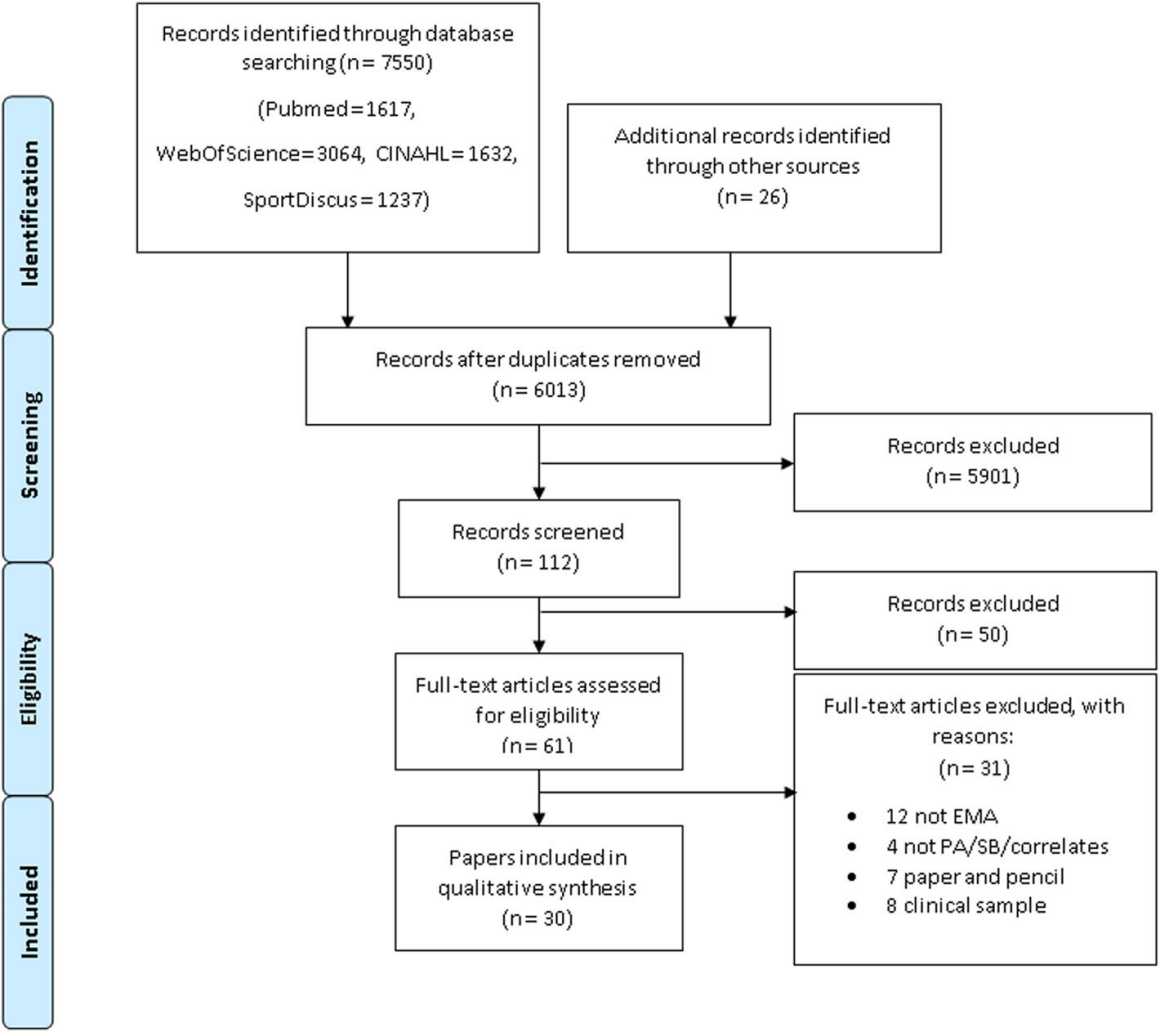
PRISMA Flow Diagram for paper selection process

The PRISMA guidelines were used to select the eligible articles [34]. The study selection initially started with 7576 papers. First, duplicates (*n* = 1563) were identified and removed electronically, using EndNote software (Clarivate Analytics, Philadelphia, USA, version X9.2, 2019). One researcher (LD) made further exclusions based upon the title (*n* = 5901). In case of doubt, the records were included in the abstract review phase, and articles were further excluded based upon the abstract (*n* = 51), which was done by one researcher (LD). In case of doubt, a second researcher (ADS) was consulted. The last exclusion was made on full text (*n* = 29). Two independent researchers (LD & ADS) reviewed all full-texts. When doubt was raised by one of the two reviewers, eligibility was discussed until consensus was reached. During all stages of the selection procedure (title, abstract and full-text), the same exclusion criteria were used. This resulted in 30 eligible studies. An overview of the study selection is provided in Fig. 1.

### Data extraction

A data extraction (coding) scheme was developed, and iteratively refined by LD, GC and ADS to ensure comprehensive data capturing. The scheme was based on the CREMAS, an adapted STROBE checklist for reporting EMA studies [33]. Variables on content validity were added based on a brainstorm session conducted by LD, GC and ADS.

Data were independently extracted by LD and ADS using a standardized form. Data was categorised within the following subdivisions:

1. study and sample characteristics (i.e. author, publication year, target population, health domain, type of study and mean sample age, and % female),
2. content validity (i.e. behaviour and correlates measured, items reported, source of items, content validation of the items)
3. EMA methodology: (3a) EMA sampling approach (i.e. sampling type, prompt frequency, rationale prompt frequency, time selection, monitoring period, number of days, source to identify event, event definition, event training and each event); (3b) data input modalities (i.e. device, retrospective assessment period, order randomization, reminder, and prompt deactivation); and (3c) EMA completion (i.e. time to complete, latency, backfilling, completion rates, incentives, training and comprehensibility). All coded variables are defined in Table 1. Consensus was used to resolve disagreement regarding the coding categories. If consensus could not be reached, inconsistencies were discussed with a third reviewer (GC). The authors were contacted only in case of ambiguities. This resulted in contacting one author about whether pencil and paper or technology was used as a platform for the EMA [35]. The author clarified this within 2 weeks.

**Table 1:** Data extraction scheme

1. **Study and sample characteristics**	
Author	The authors of the publication
Publication year	The year in which the article was published
Target population	The particular group of people that identified as the recipients of this study (demographic characteristics)
Health domain	The health domain that the EMA study focusses on (Physical activity, sedentary behaviour)
Type of study	e.g. observational, interventional, validation, feasibility
Mean sample age	The mean age of participants in the sample of the EMA study
% female	The percentage of women of the sample
2. **Content validity**	
Behaviour and correlates measured	Targeted variables measured by the EMA (e.g. activity type, activity duration, activity intensity, sitting time, number of sedentary breaks, mood, affect, intention, location, social company)
Items reported	The specific items that were used to measure the target variables are reported (yes,no).
Source of the items	The source of the items used in the EMA (e.g. pilot study with experts, pilot study with users, existing EMA questionnaire, existing non-EMA questionnaire, self-made)
Content validation of the items	Indicated if (yes, no), and by what methods, content validity of the used items was assessed (e.g. cognitive interviews end-users, cognitive interviews experts, pilot study)
3. **EMA methodology**	
**a. EMA sampling approach**	
Sampling type	The sampling strategy that is used to prompt and present the EMA questionnaire (e.g. time-based sampling, event-based sampling)
Prompt frequency	Frequency of prompts per day. Break down by weekdays and weekend days if applicable
Rationale prompt frequency	Rationale given for selecting a certain prompt frequency (e.g. variability and occurrence predictor/outcome, participant burden)
Time selection	If using time-based sampling, indicate what type of schedule is used (e.g. fixed, random, semirandom (random with restrictions e.g. time interval))
Monitoring period	The daily monitoring period, if applicable, difference between weekdays and weekend days
Number of days	The number of days the study lasted, and how many weekdays versus weekend days
Source to identify event	If event-based sampling, is the EMA self-initiated of device-initiated? (Self-initiated/Device-initiated)
Event definition	If event-based sampling, the definition that is used to identify the targeted events
Event training	If self-initiated event-based sampling, is it indicated if, and by what methods, training of participants to correctly identify events was provided?
Each event	If event-based sampling, is each event a trigger for the EMA? If not, how many events?
**b. Data input modalities**	
Device	The device that was used to prompt, present the EMA questionnaire and take the answers of the participants (e.g. mobile phone, tablet, handheld, PC)
Retrospective assessment period	The time window that participants had to reflect on during each EMA (now, right before the prompt, past amount of time (e.g. past hour), since the last entry)
Order randomization	Are the items of the EMA questionnaire presented in a randomized order? (e.g. yes, no, branching)
Reminder	Reminder after not immediately answering an EMA prompt. If so, number of reminders and timing.
Prompt deactivation	Deactivation of the EMA prompt after a certain time. If so, timing of the deactivation (yes/no)
**c. EMA completion**	
Time to complete	Time needed to complete one EMA
Latency	The amount of time elapsed between prompt signal and answering of prompt
Backfilling	Number of diaries completed in bulk at the same time
Completion rates	Total number of answered EMA prompts across all subjects and the average number of EMA prompts answered per person. Report compliance rate both by monitoring days and waves, if applicable.
Incentives	Reward provided to participants of the EMA study. If yes, what incentive was provided?
Training	Indicated if (yes, no), and by what methods, training of participants for EMA protocol was provided (if yes, e.g. test period (How long?), providing clear instructions, systematically going through the EMA items together)
Comprehensibility	Indicated if comprehensibility of the EMA protocol was tested? (yes, no) If so, by what methods is comprehensibility tested? (e.g. opportunities for questions, guided practice sessions, feedback consultation after test period)

## Results

An elaborate and detailed overview of all coded information from the included EMA studies is provided in an additional file [see Additional file 1].

### Study and sample characteristics

The study selection is outlined in Fig. 1, ‘PRISMA diagram’ [36]. In total, 30 papers met the inclusion criteria of this review. These studies represented 20 unique and independent EMA studies. Of these 20 EMA studies, 17 each resulted in a single paper, the ‘MASH’ study resulted in 2 papers [37, 38], ‘The Mobile Healthy PLACES’ study resulted in 3 papers [39, 40, 41], and ‘Project Mobile’ resulted in 8 papers [16, 42–48]. All papers were coded, but information extracted from papers derived from one EMA study were presented as one single study. Sample and study (publication year and study type) characteristics are presented at the level of the paper. All other (content validity and methodological) results are provided at the level of the independent study.

All included papers were observational papers. Twenty-eight of the thirty papers (93%) were published in the last 10 years; twenty of the thirty papers (67%) were published in the last 5 years.

Five papers (25%) used EMA within both physical activity and sedentary behaviour, and fifteen within physical activity research only (75%). No papers focused on sedentary behaviour only. The samples consisted of an adult 50+ population (3/30: 10%), a general adult population (14/30: 47%), students (2/30: 7%), adolescents (6/30: 20%), and children (4/30: 13%). One paper (1/30: 3%) did not mention the age of the target population. Study sample sizes varied from 22 to 526 participants and in all papers there was a gender mix, with the percentage of women ranging from 47 to 88% (mean proportion of women was 61% across all papers).

### Content validity

#### Behaviour and correlates measured

Before discussing content validity of the used items, we provide an overview of the variables and correlates measured by the EMA. The variables used to assess behaviour and its correlates varied between the studies. Specific variables were often not reported. For example, studies often vaguely reported that ‘current physical activity’ was assessed. However, this is not specific as it may refer to activity type, activity duration, or activity intensity. Of the studies that did provide sufficient information, activity type was assessed in 8 studies (40%) [49–56], while duration of the activity was assessed in 2 studies (10%) [49, 55]. Information on location and social company was assessed in 4 (20%) [37, 39, 57, 58] and 6 studies (30%) [37, 39, 57–60] respectively. Affect was assessed in 5 studies (20%) [39, 57, 61–63] and mood was assessed in 6 studies (30%) [58–60, 64–66]. Other correlates of behaviour, such as motivation, outcome expectancy, self-efficacy, intention, self-control, energy, fatigue and perception of enjoyment were each assessed in one study. Three studies did not use any EMA methodology to assess behaviour (physical activity and/or sedentary behaviour) [63–65]. In these studies, the behaviour was measured by accelerometry.

To describe if and how content validity of the items used in the included EMA studies was assessed, we coded the following variables: items reported (Y/N), source of the items, content validation of the items (Y/N).

#### Items reported

Only 12 of the 20 studies (55%) reported all of the specific items that were used in the EMA to measure variables such as activity type, location and presence of others, mood, affect, and motivation [37, 39, 51–53, 56, 57, 60–64]. Three studies (15%) provided some examples of the items used [58, 66, 67]. Five studies (25%) did not report the actual items, but only reported the construct that was measured in the EMA [49, 54, 55, 65, 68].

#### Source of the items

As content validity refers to the degree to which the content of an instrument is an adequate reflection of the construct to be measured, it is important that the used items measure the specific construct within a specific population using a specific assessment tool (here EMA). Therefore, the source of the items is important (e.g. pilot study with experts, pilot study with users, existing EMA questionnaire, existing non-EMA questionnaire, self-made). Only 8 (40%) of the studies reported the full source of the used items [49, 55, 57, 58, 61, 64, 68, 69]. For example, the study of Bedard et al. indicated that the items measuring affect were taken from the Positive and Negative Affect Schedule for Children, which is a non-EMA questionnaire [57]. In addition, 2 items were taken from the State Self-Control Capacity Scale, which is also a non-EMA instrument. Koch et al. used an instrument to assess mood developed by Leonhardt et al. [64, 70]. This instrument, based on the Multidimensional Mood Questionnaire, was shown to be appropriate for assessing within-subject dynamics of mood via e-diaries in everyday life [71]. Four studies (20%) provided the source of some of the items, but not all [59, 62, 63, 66]. Eight of the studies (40%) reported no source of the items [37, 39, 52–54, 56, 60, 61]. Of the ones that reported the source (*n* = 12), often not a lot of information was available. Studies mainly reported to use items from existing questionnaires. Five studies (25%) reported to have used items from an assessment tool that was specifically developed or validated for the purpose of ambulatory assessment (EMA) [49, 57, 58, 65, 66]. Two of these studies reported having adapted the existing questions [49, 57]. Eight studies (40%) used items from existing non-EMA questionnaires, such as the Positive and Negative Affect Schedule for Children, the State Self-Control Capacity Scale, the Feeling Scale, POMS-15, PANAS [55, 57–59, 61–64, 68]. Of these eight studies, one study reported making some adaptations to the questions [59]. However, as was the case with items from EMA questionnaires, specific adaptations made to items derived from non-EMA questionnaires were not reported.

#### Content validation of the items

Striking was that none of the 20 studies explicitly reported if, and by what methods content validity of the items was assessed.

### EMA methodology

#### EMA sampling approach

As stated in the inclusion criteria, all studies used EMA methodology, meaning that participants were signalled to complete assessments throughout the course of the day at random or fixed times (time-based sampling) or when exhibiting certain behaviours (event-based sampling). In this review, fourteen studies (70%) used time-based sampling [37, 39, 49, 55, 57, 58, 60, 62, 63, 66, 67, 72], two studies (10%) used event-based sampling [54, 68] and four studies (4/20: 20%) used a combination of both [52, 61, 64, 65]. The total monitoring period duration varied from 1 day to 6 months. Most of the studies (16/20: 80%) monitored for a period between 1 and 10 days. Three of the 20 (10%) studies monitored for 14 days [49, 52, 59] and one study monitored for 6 months (event-based sampling for the full 6 months and 6 weeks of time-based sampling) [61]. Of the 20 studies, 9 (45%) used a fixed monitoring period starting in the morning and ending in the evening [42, 49, 53, 57, 58, 60, 63, 65, 67]. Five studies (25%) used a different monitoring period for week and weekend days [39, 52, 56, 62, 64]. The study of Rusby et al. (2014) used a different monitoring period, depending on the day of the week [66]. One study (5%) used a continuous monitoring period, but with the instruction to turn off the prompts during sleep and temporarily during waking hours when necessary (e.g. religious services, job-related meetings) [73]. Two study (10%) individually programmed the monitoring period (time-based and event-based) based on sleep/wake/activity patterns of the participants (e.g. restricting potential times for time-based sampling tailored to the school schedule) [37, 55].

##### Time-based sampling

Of the eighteen studies that used time-based sampling or a combination of time- and event-based sampling, six (30%) used a fixed time selection (fixed prompt times) [37, 49, 55, 59, 62, 64]. For example, the study of Atienza et al. prompted users four times per day at fixed moments, i.e. 7:45 am, 11:45 am, 3:45 pm and 7:45 pm [49]. Eight (40%) studies used a semi-random time selection, meaning that they prompted at random times within predetermined time windows [39, 42, 53, 57, 58, 61, 63, 66]. For example, in the study of Maher et al., the e-diary prompted 6 times per day, once within each 2-h block of the day (i.e. 8:00 am-10:00 am, 10:00 am-12:00 pm, 12:00 pm-2:00 pm, 2:00 pm-4:00 pm, 4:00 pm-6:00 pm, 6:00 pm-8:00 pm) [53]. The study of Reichert et al. (2016) used both a semi-random and a fixed time selection by implementing 2 fixed prompts per day (8 am; 10:20 pm), and additionally at least every 100 min after the last event-based prompt, with minimum 40 min in between each prompt [65]. Three studies (15%) used a total random time selection to prompt the users. Prompt frequency in all of these 18 studies varied from 2 times to 30 times a day, but most studies (15/18: 83%) used a frequency between 2 and 10 prompts a day [39, 42, 49, 52, 53, 55–62, 64, 66]. Three studies prompted the participants more than 10 times a day [37, 63, 65]. However, in most studies (11/18: 61%) no rationale was provided for the used prompt frequency, time selection, and monitoring period. Of the seven studies in which a rationale was given, two studies (2/7: 29%) indicated that a specific frequency was chosen in order to ensure adequate spacing across the day [39, 42]. Two studies (2/7: 29%) selected a certain frequency pattern because of the occurrence pattern of physical activity [59, 66]. Rusby et al., for example, selected a 7-day monitoring period with prompts during non-school hours only, having prompts three times on Monday through Thursday (from 3:30 to 9:30 p.m.), four times on Friday (from 3:30 to 11 p.m.), six times on Saturday (11:30 a.m. to 11 p.m.), and five times on Sunday (11:30 a.m. to 9:30 p.m.). This was to accurately capture the free time activities across a full week as many activities for youth follow a weekly schedule and leisure-time activities are likely to differ between week and weekend days [74]. One study (13%) chose the prompt frequency to prevent expectancy effects [63] and one study (1/7: 13%) used a prompt frequency that was tailored to the users’ school schedules [58]. Finally, one study opted for a fixed time selection because of the preliminary nature of the study [49]. When considering both the prompt frequency and duration of the EMA, when collecting data over longer time periods, fewer assessments per day were generally used, presumably to reduce participant burden.

##### Event-based sampling

Of the six studies that used event-based sampling or a combination of time- and event-based sampling, three (50%) used a device-initiated assessment, in which the device (handheld/smartphone) initiated the assessments in response to a behaviour or event (2 GPS, 1 mobile phone’s built-in motion sensor) [52, 64, 65]. The other three (3/6: 50%) used a self-initiated assessment in which participants were asked to self-initiate assessments in response to specific behaviours or event [19, 54, 61, 68]. In one of these event-based studies (17%), it was not reported whether each event had to be assessed [65]. In the study of Koch et al., the device prompted users after each event, but users were asked to only answer 4–7 prompts per weekday. On the weekend, participants were asked to complete 8–17 prompts per day [64]. In one study (33%), each event had to be assessed, but there was a 30-min gap between the prompts to avoid excessive prompting [52]. In three studies, the self-initiated event based studies, all events had to be assessed [54, 61, 68].

Events in the *device-initiated assessments* were defined in all three studies. In two studies, it was defined as a distance covered of more than 0.5 km measured by the GPS [64, 65]. In the third study, an event was defined based on motion sensor measurements and automatically detected periods of (1) Activity (15+ minutes of high-intensity activity followed by 10+ minutes of low-intensity activity); (2) No-Activity (60+ minutes of low-intensity activity followed by 2+ minutes of moderate-intensity activity); and (3) No-Data (10+ minutes of no activity data followed by 1+ minutes of some activity data) [52]. Events in the three studies using *self-initiated assessment* were defined as follows: (1) a session of structured walking-for-exercise (i.e. not lifestyle PA) [61], (2) movement to another location + activities (e.g. sleep, eat, work) performed at each place [68], and (3) the start of a new activity in one of seven categories (sleeping/resting; personal care; eating/drinking; job; leisure time; transport; household) [54]. However, none of these studies that were using self-initiated assessments reported on providing any sort of training to the respondents to correctly identify predefined events.

### Data input modalities

The two most commonly used electronic devices to administer the EMA were smartphones and handheld computers. These handheld computers, also referred to as personal digital assistant (PDA), can be seen as the precursor of the smartphone. PDAs were largely discontinued in the early 2010s after the widespread adoption of highly capable smartphones [75]. Therefore, of the six studies that used a handheld device to provide the EMA [37, 54, 59, 61, 63, 76], five were published more than 5 years ago [37, 54, 59, 63, 76]. Thirteen studies (65%) used smartphones [39, 42, 52, 53, 55–58, 60, 62, 64, 65] and one study used an iPod Touch [66]. One study (5%) did not report the specific device that was used to administer the EMA [68].

The reference point/period upon which participants had to reflect during the EMA question, after receiving a prompt, was reported in fifteen of the twenty studies (75%). However, this is not relevant in the event-based sampling studies because in these studies participants have to reflect on the current event/activity [54, 68]. In the study of Scheers et al. for example, participants were asked to register their activities in the electronic diary each time a new activity was started [54]. In the eighteen time-based sampling studies, the retrospective assessment period was reported in fifteen studies. In six of these studies, the retrospective assessment period was reported as ‘now’ [37, 57, 60–63], in three as ‘right before the prompt’ [39, 42, 53] and in another three as ‘since the last entry’ [49, 55, 59]. Three studies reported a retrospective assessment period as ‘over the past 3 and a half hours’ [58], ‘over the past 2 h’ [56] and ‘over the past 30 minutes’ [52]. In six of the twenty studies (30%) it was possible to delay answering the prompt with for example 5, 10, 15 or 20 min [49, 59, 61, 63–65]. Nine studies (45%) provided one or more auditory reminders when participants did not answer a prompt. In 8 of the 9 studies, these reminders were given 1 to 3 times within a period of 10 min after the first prompt [37, 39, 42, 49, 52, 53, 59]. The study of Spook et al. provided reminders 30 min and 60 min after the first prompt [58]. In the study of Sternfeld et al., the program sent an automatic text message reminding the participant to record their activities if expected transmissions were not received [55]. Eight (40%) did not mention a deactivation of the prompt. Five (25%) deactivated the prompt after 1 to 3 reminders [37, 42, 52, 53, 77] and five (25%) deactivated the prompt after a fixed time window (e.g. 5 min, 20 min, 45 min) [49, 59–61, 66]. Seven of the twenty (35%) studies did report on the randomisation of the items during the assessment. Four (20%) studies worked with branching in the answering system [39, 52, 54, 58]. One study provided a pseudo-randomized order of the adjectives [62]. One study provided a random subset of variables during each assessment [57] and another study reported on presenting the items in a mixed order and with reversed polarity using seven-point Likert scales [63].

### EMA completion

As in all methodologies that follow people over time, compliance is critical. The majority of the studies (19/20: 95%) reported at least some information on completion rates [37, 39, 42, 52–56, 57–62, 64–66, 68]. The range of study methods and variations in the types of compliance data reported in our reviewed studies make it difficult to compare completion rates between studies. However, of the studies that reported an overall completion rate (13/20: 65%), the average completion rate was 77%, with individual study rates ranging from 56 to 97.7%. A potential approach to further enhance compliance is the use of incentives. In this review, 12 of the 20 studies (60%) reported to have provided some kind of incentive. Ten studies (50%) provided monetary incentives, such as cash, gift cards and coupons [37, 39, 42, 52, 53, 57, 58, 65, 66, 68]. Six of these provided additional incentive for each completed EMA entry [37, 39, 42, 52, 53, 57]. One study (5%) provided a summary of individual PA results based on the assessments [49] and one study (5%) provided course credit for students [63]. Eight studies (40%) did not report providing an incentive. However, this does not imply that no incentives were provided. Therefore, the relationship between incentive and compliance cannot be discussed here.

Twelve of the twenty studies (60%) provided information on the time to complete a single assessment. In all studies it took less than 5 min to complete an assessment. In ten studies (50%) it took between 1 and 5 min [37, 39, 53, 55–59, 66], in two (10%) less than a minute [52, 60]. Only one study (5%) provided information on the latency of filling out the assessments [51]. No study provided information on backfilling.

An important prerequisite of high compliance rate is comprehensibility of the EMA protocol. Therefore training the participants to familiarize them with the specific EMA items and EMA protocol can be a useful tool to ensure comprehensibility of the EMA and consequently increase compliance. However, only 9 of the studies (45%) provided the participants a form of training in using the EMA. Three studies (15%) implemented a test period to let the participants get used to the assessment [39, 60, 71]. For example, in the study of Emerson et al. (2017), prior to the start of the monitoring period, training on the e-diaries (i.e. researcher guided assessments and a three-day practice period) was given to the participants. Three studies (15%) organized a one-off practice assessment to familiarize users with the study protocol and the items used with the EMA [54, 40, 78]. Dunton et al. (2012) for example organized a guided practice assessment in the presence of a research staff member and participants were given the opportunity to ask questions [43]. Furthermore, three studies (15%) reported having provided some kind of training to familiarize participants with the EMA, but did not specifically mention which type of training [52, 62, 65]. For example, Maher et al. (2018) reported broadly that participants were familiarized with the study protocol and the technical equipment [52].

Of the studies that provided training, only 1 (5%) reported that user comprehensibility was assessed. A pilot study thoroughly tested all EMA items in the target population for comprehension and applicability [57].

## Discussion

This systematic review examined the use of EMA in physical activity and sedentary behaviour research. As earlier research indicated, EMA is a valuable approach to collect self-reported data on physical activity/sedentary behaviour and their correlates. This review however revealed some issues concerning the assessment and reporting of the content validity of EMA items and issues related to the substantiating and reporting of certain methodological considerations.

### Content validity

As stated in the introduction of this review, EMA is an appealing methodology for many researchers. It is characterized by brief and repeated assessment of experiences and contextual information. The development of EMA has similarities with the development of traditional questionnaires, however there are some important considerations that need to be taken into account during the development of an EMA measure. The use of long questionnaires is not advised, and items have to make sense at a specific moment in time. Items from validated questionnaires are not by default valid when used in EMA. Assessing content validity of the items, specifically for EMA is therefore strongly recommended as a first step in developing an EMA. This review shows that not much attention was given to the content validity of the items used. This may indicate that either content validity was not assessed specifically for EMA, or that it was assessed but not reported. A striking finding was that the majority of the included studies did not report the items that were used to measure a certain construct. Furthermore, the source of the items, whether training was provided to the participants and whether participants understood the items were only reported by a few studies. In general, the included studies did not report much information on content validity. Nevertheless, content validity is a quintessential, first step in the process of validation. If items turn out not to be content valid, researchers may have difficulties in interpreting the findings. Furthermore, a lack of content validity will affect all other measurement properties and may decrease construct validity, interpretability and responsiveness [81]. All items used in an EMA should be relevant for the construct of interest (within a specific population and context of use), and the EMA items should be comprehensive with respect to the users’ concerns. More specifically, to address this issue and to create unambiguity, a COSMIN guideline was recently developed with a new methodology for evaluating content validity of patient outcome measures [79]. This framework may also provide useful directions to assess content validity of EMA. As stated by the COSMIN guidelines, content validity has to be assessed by scoring the following criteria: relevance (Are the included items relevant for the construct of interest? Are the included items relevant for the target population of interest? Are the included items relevant for the context of use of interest? Are the response options appropriate? Is the recall period appropriate?), comprehensiveness (Are all key concepts included?) and comprehensibility (Are the instructions understood by the population of interest as intended? Are the items and response options understood by the population of interest as intended? Are the items appropriately worded? Do the response options match the question?). Although the coded content validity variables in this review do not perfectly match these 3 criteria, this review revealed that the existing EMA studies mainly make efforts to meet the comprehensibility criteria, but they neglect the relevance and comprehensiveness criteria. Within the included EMA studies, it is often easily assumed that all the items are relevant to the construct to be measured. In addition, in order to minimize the burden on the participant, EMA studies aim to make assessments as short as possible. This entails the danger that some key aspects of the construct could be missing. As stated earlier, the fact that in this review very few studies reported on content validity does not necessarily mean that content validity was not assessed. Therefore, it may be recommended in the future to report more carefully on the content validity in EMA studies and to add reporting or rating guidelines for content validity to the CREMAS checklist for EMA studies [33]. An adapted CREMAS checklist was added in an additional file [see Additional file 2].

### EMA methodology

A second aim was to document critical features of EMA methodology in terms of EMA sampling approach, data input modalities and EMA completion. Liao et al. developed the CREMAS checklist as a framework to report EMA methodology in a clear and uniform way. This review allowed giving an update on the reporting of EMA methodology using this CREMAS checklist.

Most studies applied time-based sampling. In the field of physical activity and sedentary behaviour, this form of sampling is technically less challenging than event-based sampling. These two sampling strategies provide different insights: the time-based strategy usually aims to acquire representative characteristics and patterns of physical activity and sedentary behaviour across time, whereas an event-based sampling strategy is often used to examine correlates of physical activity and sedentary behaviour [22]. On the basis of the study rationales, variations of time-based (e.g. prompting participants at random times and within a window of time) and event-based (e.g. participant self-initiated self-report in response to occurrence of specific events or device-initiated events, such as location via Global Positioning System (GPS), or bouts of physical activity via accelerometer) strategies have been used, either on their own or in combination. The rationale for choosing a certain sampling strategy needs to be reported. Furthermore, the time-based sampling modalities (time selection, prompt frequency, monitoring period, number of days) were very diverse in the included studies. An earlier review on EMA in diet and physical activity research in youth revealed a mean of 7 assessments per day [33], which is similar to the current review finding showing a mean of 5 assessments per day across the studies. Diverse sampling modalities may possibly be related to diverse constructs and research questions. For example, few assessments may be enough to examine group differences in average physical activity / sedentary behaviour levels, but more assessments per day are typically required to adequately capture dynamic within-person processes such as affective and cognitive factors related to physical activity and sedentary behaviour [80]. It is not possible to provide specific guidelines on sampling modalities. However, in this review we want to emphasize the need to report these sampling modalities and their rationale. In sum, it appears that potential improvements for time-based EMA sampling may lie in balancing the number of prompts with the level of detail required for varying degrees in fluctuating variables.

Among the 6 event-based EMA studies reviewed, only three studies used a device-initiated assessment [52, 64, 65]. Currently, the majority of the mobile EMA studies operationalized event-based sampling using a participant self-initiated self-report approach. However, information obtained using this self-initiated approach may be subject to systemic under- or over-reporting [81]. Self-initiated event sampling also requires a large responsibility of the user to start the assessment when an event occurs, which may result in more missing values. Furthermore, an important aspect of self-initiated event-based approaches is providing a clear definition of the event of interest upon which users have to report, and offering training for event identification. None of the studies reported any event training for the users. Providing training and reporting on how this training took place may further contribute to the EMA evidence-base.

An important aspect of EMA is the fact that it allows to assess physical activity / sedentary behaviour and their correlates in the moment. In order to ensure this, several methodological aspects need to be taken into account. For example the use of mobile technology facilitates this because of the constant physical availability of the devices. In this review, mostly handhelds and smartphones were used to present the EMA. Studies using a handheld (PDA) were mainly conducted between 2005 and 2010. More recent studies used smartphones. Only one study used a smartwatch to conduct the EMA. The constant physical availability of a smartwatch (smartwatches are worn on the wrist) however allows for instant accessibility and is therefore perfectly suited for EMA minimizing user burden due to interruption of activities [82]. Future research may wish to further explore the potential of smartwatches to conduct EMA. In this review most studies reported the reference point for the EMA, with the majority using a retrospective assessment period stated as ‘now’, ‘right before the prompt’. More than half of the studies did not report using any reminders or deactivating the prompts. Therefore, it is important to use a limited number of reminders soon after the first prompt and to eventually allow the possibility to deactivate the prompt. This will avoid late answering to the prompt and backfilling all previous prompts at once in order to ensure assessments ‘in the moment’.

To fully use the potential of EMA protocols, high participant completion rates are important. Missing data often lead to a lower representativeness of the data and lower generalizability of study findings. Recognizing the importance of participant completion in EMA designs, researchers should report data on completion in detail. However, in more than one third of the studies included in this review, completion rates were not reported at all. The average completion rate across studies that did report it was 76.0%. This is in line with earlier research that found average completion rates of 71 and 86% [33, 83]. These relatively high compliance rates indicate that participants are willing to take on the burden of frequent assessment necessitated by EMA. This may be due to the incentive (often monetary) that was provided in the majority of the studies, however this link cannot be demonstrated in this review as some studies may have provided incentives, without reporting it.

This review revealed an increasing number of studies using EMA in physical activity research. In sedentary behaviour research however, the use of EMA is not yet established. Furthermore, this review revealed that of the existing EMA studies in the field of physical activity and sedentary behaviour, few provided a rationale for using EMA methodologies. Similar to the use of EMA in other research domains (e.g. pain, mood disorders), it can only be assumed that EMA methodologies are uniquely suited to examine temporal relationships between physical activity /sedentary behaviour and affective, cognitive and other behavioural factors [83, 84]. On the other hand, this review reveals that the heterogeneity of several methodological aspects of the included EMA studies indicates that EMA methodologies are complex and various design decisions are required for their implementation. This review is unique because it is the first to examine the reporting of content validity in EMA studies on physical activity and sedentary behaviour. However, the present review also has some limitations. First, because of practical reasons, independent double coding of titles and abstracts was not done during study selection. Although we attempted to be complete in the literature search, it is possible that some studies may have been missed. Second, the selection of content validity variables for the coding scheme was based on a brainstorm session and own experiences of the researchers (LD, ADS, GC). This without explicitly relying on earlier research on content validity, which could make it difficult to replicate this review study from scratch.

The following conclusions and recommendations can be made. The content validation of the EMA items was seldom reported. Therefore, before conducting EMA studies in physical activity and sedentary behaviour research, content validation is required. The COSMIN checklist provides a clear framework to do this by taking into account the three main criteria for content validity: relevance, comprehensibility and comprehensiveness. Further, this review revealed that, despite the existing CREMAS checklist, reporting on the EMA methodology did not happen in a clear and uniform way. In addition, often no rationale was provided for several methodological decisions. Therefore, three recommendations concerning EMA methodology are made. First, it is important to provide a rationale for choosing a certain sampling strategy and certain sampling modalities (time selection, prompt frequency, monitoring period, number of days). Second, to ensure assessment ‘in the moment’, it is important to carefully select the retrospective assessment period upon which participants have to reflect during the assessment, to implement a few reminders soon after the first prompt, and to deactivate the prompt after a few reminders to minimize latency and avoid backfilling. Third, as high completion rates are essential for representativeness of the data and generalizability of study findings, reporting these completion rates is recommended.

## Supplementary information

**Additional file 1:** Table with coded information from the included studies.

**Additional file 2:** Adapted CREMAS checklist.

**Additional file 3:** PRISMA Checklist.

## Data Availability

All data generated during this systematic review are included in this published article (and its additional file).
