# Supplementary material for: Content validity and methodological considerations in ecological momentary assessment studies on physical activity and sedentary behaviour: a systematic review
Source: Int J Behav Nutr Phys Act. 2020 Mar 10;17:35. doi: 10.1186/s12966-020-00932-9 (PMC7063739; doi:10.1186/s12966-020-00932-9)
Supplement: Supplementary file 1 — Additional file 1: Table with coded information from the included studies. [file 12966_2020_932_MOESM1_ESM.docx]

# Additional file 1

| **Study and sample characteristics** | | | | | | | | |
| --- | --- | --- | --- | --- | --- | --- | --- | --- |
| **Nr** | **Author** | **Publication year** | **Target Population** | **Health Domain** | **Type of Study** | **Mean Sample Age** | **% Female** |  |
| 1 | Atienza et al. | 2006 | Adults Age 50+ | PA | Observational | 60.9 | 60% |  |
| 2 | Bedard et al. | 2017 | First year University student | PA | Feasibility | 18.3 | 47% |  |
| 3 | Dunton et al. | 2009 | Adults Age 50+ | PA | Observational | 60.7 | 70% |  |
| 4 | Dunton et al. (MASH) | 2007 | Adolescents | PA | Observational | 14.5 | 49% |  |
| 5 | Dunton et al. (MASH) | 2005 | Adolescents | PA | Observational | 14.5 | 49% |  |
| 6 | Dunton et al. (Mobile Healthy PLACES) | 2011 | Children 9-13yo | PA | Observational | 11.0 | 58% |  |
| 7 | Dunton et al. (Project Mobile) | 2012 | Adults | PA and SB | Observational | 40.4 | 73% |  |
| 8 | Dunton et al. (Project Mobile) | 2015 | Adults | PA | Observational | 40.5 | 72% |  |
| 9 | Dunton et al. | 2016 | High School students | PA | Observational | 15.9 | 54% |  |
| 10 | Dunton et al. (Mobile Healthy PLACES) | 2014 | Children 9-13yo | PA | Observational | 10.9 | 58% |  |
| 11 | Emerson et al. | 2017 | Adults 18-65yo | PA | Observational | 47.7 | 88% |  |
| 12 | Engelen et al. | 2016 | Adults | PA | Observational | Not reported | 50% |  |
| 13 | James et al. | 2016 | Not mentioned | PA | Observational | 64% over age of 34 | 67% |  |
| 14 | Jones et al. (Project Mobile) | 2017 | Adults | PA | Observational | 40.3 | 72% |  |
| 15 | Koch et al | 2018 | Adolescents | PA | Observational | 15.02 | 48% |  |
| 16 | Liao et al. (Mobile Healthy PLACES) | 2014 | Children (9-13y of age) | SB | Observational | Range 9-13y | 49% |  |
| 17 | Liao et al. (Project Mobile) | 2015 | Adults (27-73y of age) | PA and SB | Observational | Not reported | 73.5% |  |
| 18 | Liao et al. (Project Mobile) | 2017a | Active adults | PA | Observational | 39.8 | 73% |  |
| 19 | Liao et al. (Project Mobile) | 2017b | Low-active adults | Pa | Observational | 40.4 | 72.5% |  |
| 20 | Maher et al. (Project Mobile) | 2018 | Older Adults | PA and SB | Validation/Feasibility | 72 | 63% |  |
| 21 | Maher et al. | 2017 | Adults | PA | Observational | 40.3 | 74.2% |  |
| 22 | Nierman et al | 2016 | Adults | PA | Observational | 43.8 men; 45.2 women | 66% |  |
| 23 | Pickering et al. (Project Mobile) | 2016 | Adults | PA | Observational | 40.3 | 72.4% |  |
| 24 | Reichert et al | 2016 | Adults | PA | Observational | 23.4 | 62.4% |  |
| 25 | Rusby et al | 2014 | Adolescents | PA and SB | Observational | 7th grader | 51% |  |
| 26 | Scheers | 2012 | Adults | PA and SB | Observational | 41.4 | 52% |  |
| 27 | Schwerdtfeger et al. | 2010 | Adults | PA | Observational | 31.7 | 52 |  |
| 28 | Spook et al | 2013 | Adolescents | PA | Validation/Feasibility | Range 16-21 | 57% |  |
| 29 | Sternfeld et al | 2012 | Adults | PA and SB | Validation | 55,6 | 52,5% |  |
| 30 | Zink et al. | 2018 | Children | PA | Observational | 9.6 | 51% |  |
|  |  |  |  |  |  |  |  |  |

| **Content Validity** | | | | | |
| --- | --- | --- | --- | --- | --- |
| **Nr** | **Author** | **Behaviour and correlates measured** | **Items reported** | **Source of the items** | **Content validation of the items** |
| 1 | Atienza et al. | Whether they have been PA, which of 12 activities they performed and the number of minutes for which they performed each activity. | No | Adapted from prior studies focused on momentary assessments using pocket computers (Atienza, Collins, & King, 2001; King, Oka, & Young, 1994) | Not reported |
| 2 | Bedard et al. | Information on current activity, physical location, type of social company, acute outcome expectancy, barrier self-efficacy, intentions, current affective and feeling states, as well as state motivation to be active and self-control | Yes (can be found in a previous publication (61)) | Most of the items have been used in previous research and have been found to be valid measures (33, 48, 69, 87)+ based on the Positive and Negative Affect Schedule for Children +2 items from the State Self-Control Capacity Scale (non-EMA) | Some of the items were validated in previous EMA research, but not within this population. Other items were taken (and adapted) from non EMA questionnaires. |
| 3 | Dunton et al. | Physical activity, self-efficacy, mood, perceived situational control and demand, energy, fatigue, social interactions, and stressful events. | Not all the items (some examples) | Self-efficacy: adapted from previous measurement tools (non EMA)(88)/ rest: not mentioned | Not reported |
| 4 | Dunton et al. | Current activity, social company, current location | Yes (screenshots) | Not reported | In a previous study, differences in physical activity intensity among  diary-reported exercise, walking, and nonphysical activities were validated with heart-rate monitoring and accelerometry (45) |
| 5 | Dunton et al. | Main activity type, physical location, distance from home, social company, positive affect, negative affect and enjoyment | Yes | Not reported | Not reported |
| 6 | Dunton et al. | Activity type | Yes  (screenshots) | Not reported | One of the aims of this study was to evaluate the criterion validity of the EMA self-reports of physical activity and sedentary behavior by comparing with time-matched data collected through an accelerometer. |
| 7 | Dunton et al. | Major activity type, methods of carrying the mobile phone, reasons for not carrying the mobile phone, and other psychological and contextual factors related to behavior | Yes (screenshots) | Not reported | Not reported |
| 8 | Emerson et al. | Structured walking-for-exercise (self-initiated even-based) and incidental affect (time-based) | Yes | Incidental affect: the Feeling Scale (non-EMA questionnaire) | The Feeling Scale has been used to measure incidental affect in numerous prior studies of PA (non-EMA) (89) and has been shown to be related to other self-report measures of incidental affect (90) |
| 9 | Engelen et al. | Posture, musculoskeletal issues, task, social interactions, mood and perceptions of engagement and productivity | Yes | Not reported | Not reported |
| 10 | James et al. | Travel/activity | No, participants recorded departure and arrival times, and mode of travel for each trip. At each destination, participants recorded activities options from lists. | Based on sun exposure/protection logs developed in previous research.(91) | Not reported |
| 11 | Koch et al | Mood (three dimensional construct: good–bad, alertness–tiredness, and calmness–tension)  Non-exercise activity: triaxial acceleration  sensor (move-II or move-III) | Yes | The assessment of mood was realized by using an instrument developed by Leonhardt et al (92). This instrument, based on the Multidimensional Mood Questionnaire (93) | Earlier research (92) showed this instrument to be appropriate for assessing within-subject dynamics of mood via e-diaries in everyday life |
| 12 | Maher et al. | Activity type | Yes  (screenshots) | Not reported | Not reported |
| 13 | Nierman et al | Affect, MVPA (only evening) | Yes | Affect: POMS-15, two subscales, ‘vigor’ and ‘fatigue’ (non EMA)  MVPA: not reported | Not reported |
| 14 | Reichert et al. | Mood (two bipolar items for the mood dimensions valence, energetic arousal, and calmness)  Non-exercise activity: triaxial acceleration  sensors of the move-II2 | No | This instrument was based on the Multidimensional Mood Questionnaire (94) | This instrument was developed and evaluated for the purposes of ambulatory assessment studies (93) |
| 15 | Rusby et al | Activities, mood and peer present. | Not all (some examples) | Similar EMA mood state measures have been validated with middle school youth (95)  Activities, peer present: not reported | Mood state measure have been validated with middle school youth (95) |
| 16 | Scheers et al | Activity type (7 main categories) | No | Not reported | Not reported |
| 17 | Schwerdtfeger et al. | Positive and negative affective states (throughout the day, in the evening), typicality of the day  Bodily movement: uniaxial  accelerosensors (Actigraph GT1M) | Throughout the day: yes, 14 adjectives were presented (rated on 5 point Likert scale)  In the evening: yes, participants were asked to rate how they felt during the recording day | **Assessment throughout the day:** Not reported  **Assessment in the evening**: PANAS (non EMA) | Not reported |
| 18 | Spook et al | **PA condition**: mood, sedentary behavior, physical activity, need for physical activity, evaluative emotions, location, social context, behavior intention, active transport, possible barriers, and feelings of security | Not all (some examples) | Previous EMA and non-EMA PA questionnaires (46, 96-101) | Yes, feasibility and usability assessed after use |
| 19 | Sternfeld et al | Activity type, activity intensity and activity duration. | Not specific | Based on a compendium of physical activities (not EMA)(102) | Not reported |
| 20 | Zink et al. | Activity type | Yes | Not reported | Not reported |

| **EMA sampling approach** | | | | | | | |
| --- | --- | --- | --- | --- | --- | --- | --- |
| **Nr** | **Author** | **Sampling type** | **Prompt frequency** | **Rationale prompt frequency** | **Time selection (time-based Sampling)** | **Monitoring period** | **Number of days** |
| 1 | Atienza et al. | Time-based sampling | 4 per day (7:45am;11:45am;3:45pm;7:45pm) | Fixed interval because of preliminary nature of the study | Fixed | 7:45am-7:45pm | 14 days (2 weeks) |
| 2 | Bedard et al. | Time-based sampling | 7 per day (every 2 hours) | Not reported | Semirandom (predetermined time intervals) | 9:00am-11:00pm | 5 days |
| 3 | Dunton et al. | Time-based sampling | 4 (7:45am;11:45am;3:45pm;7:45pm) | Capturing physical activity bouts occurring throughout the day (morning, midday, afternoon, evening), while minimizing recall errors associated with longer assessment windows | Fixed | 7:45am-7:45pm | 14 days (2 weeks) |
| 4 | Dunton et al. | Time-based sampling | 25 to 30 per day (every 30 ± 10 minutes) | Not reported | Fixed | Programmed individually based on wake/sleep patterns | 9 waves of 4 days (2 twice a year between 9th and 12th grade) |
| 5 | Dunton et al. | Time-based sampling | 3 weekday (4-6pm;6-8pm;8-8:30pm)  7 weekend day (8:30-10am;10am-12pm;12-2pm;2-4pm;4-6pm;6-8pm;8-8:30pm) | Leisure time PA, allow for adequate spacing across each day | Semirandom (predetermined time intervals) | weekday (8u30am-8u30pm)/weekend day (4u30pm-8u30pm) | 4 days |
| 6 | Dunton et al. | Time-based sampling | 8 per day (6:30am;8-10am;10am-12pm;12-2pm;2-4pm;4-6pm;6-8pm;8-10pm) | In order to ensure adequate spacing across the day | Semirandom (predetermined time intervals) | 6:30am-10pm | 4 days |
| 7 | Dunton et al. | Event-based sampling (CS-EMA) and time-based sampling (R-EMA) | **Time-based EMA**: 3 per weekday (3-9pm), 7 per weekend day(7am-9pm) (non-school days) | Not reported | Random | week: 3-9pm  weekend: 7am-9pm | 14 days |
| 8 | Emerson et al. | Event-based sampling and time based sampling | **Time-based EMA:** 8 per day (completing a report each morning, after which the e-diary prompted once within each 3-h block of the day (i.e. 9:00 am-12:00 pm, 12:00 pm–3:00 pm …) | Not reported | Semirandom (predetermined time intervals) | 24 hours/day (instructed to turn of prompts during sleep) | event sampling: 6months/time sampling: week 1-4, 12 and 25 |
| 9 | Engelen et al. | Time-based sampling | 4 per day | Not reported | Random | 9am-5pm | 5 days |
| 10 | James et al. | Event-based sampling | Not reported: only event-based sampling |  |  | Not reported | 5 days (2 weekend days and 3 weekdays) |
| 11 | Koch et al | Event-based sampling and time based sampling | **Time based EMA**: 2 per day (at 4:30pm and 8:30pm)  **Event-based EMA:** minimum period between two prompts was 37min and the max 77min**.** | Not reported | Fixed | 4-8:30pm on weekdays;  8am-8:30pm on weekend days | 7 days |
| 12 | Maher et al. | Time-based sampling | 6 prompts per day (within six pre-programmed windows of 2h) | Not reported | Semirandom (predetermined time intervals) | 8am - 8pm | 10 days |
| 13 | Nierman et al. | Time-based sampling | 3 per week day (6:30am; 4:30pm; 9pm)  2 per weekend day (8am; 9pm) | Not reported | Fixed | week: 6:30am-9pm  weekend 8am-9pm | 7 days |
| 14 | Reichert et al. | Event-based sampling and time-based sampling | between 9-22 times per day:  **Time-based EMA:** 2 fixed per day (8am; 10:20pm), and additionally at least every 100min after the last event-based prompt, with min 40 min in between each prompt.  **Event based EMA:** GPS triggered | Not reported | Fixed + Semirandom (predetermined time intervals) | 7:30am-10:30pm | 7 days |
| 15 | Rusby et al. | Time-based sampling | 3 per day on Mon-Thu  4 per day on Fri  6 per day on Sat  5 per day on Sun  (Measurement occasions were randomized within 90- to 120-minute blocks and were at least 30 minutes apart) | To capture rhythms of participants' freetime (non-school) PA | Semirandom (predetermined timeframes) | Mon-Thu: 3.30-9.30pm Fri 3.30-11pm  Sat: 11.30am-11pm  Sun: 11.30am-9.30pm | 28 (4 waves of 7 days) |
| 16 | Scheers et al. | Event-based sampling | Not reported: only event-based sampling |  |  | Not reported | 7 days |
| 17 | Schwerdtfeger et al. | Time-based sampling | Every hour with a random component of 15 minutes | to prevent expectancy effects | Semirandom (predetermined timeframes) | Between 9-10am (after being familiarized with study protocol and technical equiment) until 10pm | 1 day (weekday) |
| 18 | Spook et al. | Time-based sampling | 5 times per day (8am; 12pm; 3:30pm; 6:30pm; 9:30pm, with a range of 30 minutes) | Tailored to the schedules of their schools | Semirandom (predetermined timeframes) | 8am-10pm | 7 days |
| 19 | Sternfeld et al. | Time-based sampling | 3 times per day (9am; 6p; before bed) | Not reported | Fixed | 9am - before bed | Min 4 days, max 8 days |
| 20 | Zink et al. | Time-based sampling | Up to 7 per week day ( between 3-8pm)  Up to 3 per weekend day (between 7am-8pm)  Day 1: only prompts after 5pm  Day 8: only prompts until 5pm | Not reported | Random | week: 3-8pm  weekend: 7am-8pm | 8 days |

| **EMA sampling approach** | | | | | |
| --- | --- | --- | --- | --- | --- |
| **Nr** | **Author** | **Source to identify event** | **Event definition** | **Event training**  **(only self-initiated)** | **Each event** |
| 1 | Atienza et al. |  |  |  |  |
| 2 | Bedard et al. |  |  |  |  |
| 3 | Dunton et al. |  |  |  |  |
| 4 | Dunton et al. |  |  |  |  |
| 5 | Dunton et al. |  |  |  |  |
| 6 | Dunton et al. |  |  |  |  |
| 7 | Dunton et al. | device-initiated | **mobile phone’s built-in motion sensor**: (1) Activity (15+ minutes of high-intensity activity followed by 10+ minutes of low-intensity activity); (2) No-Activity (60+ minutes of low-intensity activity followed by 2+ minutes of moderate-intensity activity); and (3) No-Data (10+ minutes of no activity data followed by 1+ minutes of some activity data) |  | Yes, but 30-minute gap between all prompts to avoid excessive prompting |
| 8 | Emerson et al. | self-initiated | Structured walking for exercise behavior (i.e. not lifestyle PA) | Not reported | Yes |
| 9 | Engelen et al. |  |  |  |  |
| 10 | James et al. | self-initiated | Movement to another location + activities (eg, sleep, eat, work) performed at each place | Not reported | Yes, participants recorded departure and arrival times, and mode of travel for each trip |
| 11 | Koch et al | device-initiated | **GPS**: when passing over a distance of 0.5 km |  | On weekdays, participants were asked to answer 4–7 prompts per day. On the weekend, participants were asked to complete 8–17 prompts per day. |
| 12 | Maher et al. |  |  |  |  |
| 13 | Nierman et al |  |  |  |  |
| 14 | Reichert et al | device-initiated | **GPS:** distance covered of more than 0.5 km |  | Not reported |
| 15 | Rusby et al |  |  |  |  |
| 16 | Scheers et al | self-initiated | each time a new activity started in one of 7 categories (sleeping/resting; personal care; eating/drinking; job; leisure time; transport; household) | Not reported | Yes, each time a new activity started |
| 17 | Schwerdtfeger et al. |  |  |  |  |
| 18 | Spook et al |  |  |  |  |
| 19 | Sternfeld et al |  |  |  |  |
| 20 | Zink et al. |  |  |  |  |

| **Data input modalities** | | | | | | | | |
| --- | --- | --- | --- | --- | --- | --- | --- | --- |
| **Nr** | **Author** | **Device** | **retrospective assessment period** | **Order randomization** | **Delay possible** | **Reminder** | **Prompt deactivation** |  |
| 1 | Atienza et al. | Handheld (Cassiopeia E-125) | Since the last entry | Not reported | Yes, 20 min | Yes, 2 out of 3 groups (auditory 10 min after prompt) | Yes, after 45min window |  |
| 2 | Bedard et al. | Smartphone (own) | Now | Not reported | Not reported | Not reported | Not reported |  |
| 3 | Dunton et al. | Handheld (Cassiopeia E-125) | Since the last entry | Not reported | Yes, 20 min | yes (auditory signal 10min later) | Yes, after 45min window |  |
| 4 | MASH | Handheld  (PalmOne) | Now | Not reported | Not reported | yes (3 reminders with 5min interval) | Yes, after three reminder signals at 1-min intervals |  |
| 5 | Mobile Healthy PLACES | Smartphone  (HTC Shadow) | Right before the prompt | Yes, random subset of variables assessed each survey (40% planned missing) | Not reported | yes (3 reminders with 1min interval) | Not reported |  |
| 6 | Project Mobile | Smartphone  (HTC Shadow) | Right before the prompt | Not reported | Not reported | yes (3 reminders with 1min interval) | Yes, after three reminder signals at 5-min intervals |  |
| 7 | Dunton et al. | Smartphone  (own/LG Nexus 4) | Over the last 30 minutes | Yes, branching sequence was only initiated in a randomly programmed 40% of surveys | Not reported | yes (2 reminder prompts at 3 minute intervals) | Yes, after two reminder signals at 3-min intervals |  |
| 8 | Emerson et al. | Handheld  (HP IPAQ v.111) | Now | Not reported | Yes, 20 min | Not reported | Yes, 20 min after notification alert |  |
| 9 | Engelen et al. | Smartphone (own) /iPod Touch | Now | Not reported | Not reported | Not reported | Yes, 5 minutes after notification alert |  |
| 10 | James et al. | Not reported | *Event-based* | Not reported | Not reported | Not reported |  |  |
| 11 | Koch et al | Smartphone (Motorola MotoG) | Not reported | Mixed order | Yes, 5,10 or 15 min | Not reported | Not reported |  |
| 12 | Maher et al. | Smartphone (MotoG4) | Right before the prompt | Branching | Not reported | yes (3 reminder signals with 5min interval) | Yes, after three reminder signals at 5-min intervals |  |
| 13 | Nierman et al | Smartphone (Samsung GT-I9001) | Affect: now  MVPA: over the last day | Not reported | Not reported | Not reported | Not reported |  |
| 14 | Reichert et al | Smartphone (Motorola MotoG) | Not reported | Not reported | Yes, 5,10 or 15 min | Not reported | Not reported |  |
| 15 | Rusby et al | iPod Touch | Not reported | Not reported | Not reported | Not reported | Yes, students had to respond within 8 min after the prompt |  |
| 16 | Scheers | Handheld (Palm Z22) | *Event-based* | Not reported | Not reported | Not reported |  |  |
| 17 | Schwerdtfeger et al. | Handheld  (Palm Zire31) | Now | Yes, pseudorandomized order of adjectives. | Yes, initialize PDA later when more appropriate | Not reported | Not reported |  |
| 18 | Spook et al | Smartphone (own) | Over the last 3,5 hours | Branching | Not reported | Yes, one after 30min and one after 60 min | Yes, counted as 'missing' after two reminders (30min/60min) |  |
| 19 | Sternfeld et al | Smartphone (own) | Since the last entry | Branching | Not reported | Yes, automatic text message reminding to record activities | Not reported |  |
| 20 | Zink et al. | Smartphone (own/MotoG) | Over the last 2 hours | Not reported | Not reported | Not reported | Not reported |  |

| **EMA completion** | | | | | | | | |
| --- | --- | --- | --- | --- | --- | --- | --- | --- |
| **Nr** | **Author** | **Time to complete** | **Latency** | **Backfilling (only when prompt is not deactivated)** | **Completion rates** | **Incentives** | **Training** | **Understanding** |
| 1 | Atienza et al. | Not reported | Not reported |  | 74% | Summary of some of their individual results based on their study data | Yes, participants were individually instructed on how to use the electronic diaries, completed a practice assessment, and were provided with a project telephone number to call in case of any technical difﬁculties experienced. | Not reported |
| 2 | Bedard et al. | Approximately 1 to 2 minutes | Not reported | Not reported | The average percentage of prompts that participants with minimum compliance (64%) responded to was 56% | $10 Starbucks gift card + $1 gift card for each prompt completed (max 5/day) | No, participants were only instructed to contact the study team if they encountered any issues with the EMA app | Not reported |
| 3 | Dunton et al. | Approximately 2-4 minutes | Not reported |  | 76% | Not reported | Not reported | Not reported |
| 4 | Dunton et al. | Approximately 1 minute | Not reported |  | The overall average daily electronic diary compliance was 82.5% | $20 for each day + $5 for each completed diary entry | Not reported | Not reported |
| 5 | Dunton et al. | Approximately 2-3 minutes | Not reported | Not reported | 80,3% | $20 plus an additional $1 for each completed EMA entry over the 4 days (up to $40 total) | Not reported | Yes,  all EMA items were thoroughly pilot  tested in the target population for  comprehension and applicability. |
| 6 | Dunton et al. | Approximately 2-3 minutes | Not reported |  | 82% | $18 plus an additional $1 for each completed EMA survey entry (32 total) over the 4-days. | Yes, participants completed a practice assessment in the presence of a research staff member and were given the opportunity to ask questions. | Not reported |
| 7 | Dunton et al. | On average, surveys that were completed without reprompting were completed in 53.2 seconds (SD=47.27, range: 8-408) | 82.3% of the surveys responded to after the first prompt,  11.0% after the first reprompt (3 minutes later)  6.7% after the second reprompt (6 minutes later) |  | Average compliance: **For CS-EMA prompts** (event-based)=84.8%,  **For R-EMA prompts** (time-based)=78.8% | up to $180 for completing the study | Not reported | Not reported |
| 8 | Emerson et al. | Not reported | Not reported |  | Time-based prompts: 88% | Not reported | Yes, training on the e-diaries took place prior to the start of the monitoring period and included researcher guided assessments and a three-day practice period. | Not reported |
| 9 | Engelen et al. | The average completion time for the survey was around 50s at the ﬁrst survey; this was reduced to 24s by around the ﬁfth survey, and remained stable throughout the later surveys. | Not reported |  | 58% | Not reported | Yes, research guided assessments and a three-day practice period | Not reported |
| 10 | James et al. | Not reported | Not reported | Not reported |  | $40 gift card | Not reported | Not reported |
| 11 | Koch et al | Not reported | Not reported | Not reported | 82% | Not reported | Not reported | Not reported |
| 12 | Maher et al. | 2-3 minutes | Not reported |  | 92% | participants answering at least 80% of the EMA prompts earning the full $80 | Yes,  Participants were familiarized with the study protocol and the technical equipment | Not reported |
| 13 | Nierman et al | Not reported | Not reported | Not reported | 97,7% | Not reported | Not reported | Not reported |
| 14 | Reichert et al | Not reported | Not reported | Not reported | 81,2% | Monetary compensation, no amount reported | Not reported | Not reported |
| 15 | Rusby et al | 2-5 minutes | Not reported |  | On average, participants completed 75 measurement occasions out of 108 possible (69%) | Monetary incentive, no amount reported | Yes, training students in using the EMA device occurred at the participating students’ school | Not reported |
| 16 | Scheers et al | Not reported | Not reported |  | Compliance for wearing the SenseWear and completing the diary was very high and only subjects with at least six days with a minimum of 22 hours and 48 min (95% of 24 hours) of data were included in the analysis | Not reported | Not reported | Not reported |
| 17 | Schwerdtfeger et al. | Not reported | Not reported | Not reported | Not reported | Course credit when applicable | Yes, participants were familiarized with the study protocol and the technical equipment | Not reported |
| 18 | Spook et al | The overall mean completion time per response was 138.7 seconds (SD 65.6) | Not reported |  | Thirty participants started using the mEMA app at Day 1 (100%), and 7 days later, 14 students still participated (44%). Compliance decreased 56%. | Participatory incentives of ten €20 coupons were randomly distributed to the students | Yes, students were able to use the app during the first day to get familiar with it | Not reported |
| 19 | Sternfeld et al | 5 min | Not reported | Not reported | 76,6% | Not reported | Yes, participants were trained in the use of the PA diaries by study staff | Not reported |
| 20 | Zink et al. | 2-3min | Not reported | Not reported | 75,7% | Not reported | Not reported | Not reported |
