# Supplementary material for: Content validity and methodological considerations in ecological momentary assessment studies on physical activity and sedentary behaviour: a systematic review
Source: Int J Behav Nutr Phys Act. 2020 Mar 10;17:35. doi: 10.1186/s12966-020-00932-9 (PMC7063739; doi:10.1186/s12966-020-00932-9)
Supplement: Supplementary file 2 — Additional file 2: Adapted CREMAS checklist. [file 12966_2020_932_MOESM2_ESM.docx]

# Additional file 2

| Title | 1 | Include ecological momentary assessment in title and key words |
| --- | --- | --- |
| Introduction | | |
| Rationale | 2 | Briefly introduce the concept of EMA and provide reasons for utilizing EMA for this study or topic of interests (eg, to examine time-varying predictors of unhealthy eating occasions in children’s daily lives) |
| Methods | | |
| Training | 3 | Indicate if, and by what methods, training of participants for EMA protocol was used |
| Technology | 4 | Describe what technology, if any, was used. Include the following information: device (eg, mobile phone, portable computer), model (eg, Nexus 4, iPod), operating system (eg, Android, Windows), and EMA program name |
| Wave duration | 5 | State the number of waves for the study (eg, 2 monitoring periods over the course of 1 year) |
| Monitoring period | 6 | State the number of days each wave of the study lasted, and how many weekdays versus weekend days |
| Prompting design | 7 | Indicate the prompting strategy used for the study (eg, event-based, interval-based, or a combination of the two). If using interval-based strategy, indicate what type of schedule is used (eg, fixed, random, or hybrid interval) |
| Prompt frequency | 8 | Intended frequency of prompts per day. Break down by weekdays and weekend days if applicable |
| Design features | 9 | Describe any design feature to address potential sources of bias (eg, reactivity) or participant burden (eg, EMA questions appearing in different orders) |
| *Items* | *10* | *Report all the specific items that were used to measure the target variables* |
| *Source of the items* | *11* | *Report the source of the items used in the EMA (e.g. pilot study with experts, pilot study with users, existing EMA questionnaire, existing non-EMA questionnaire, self-made)* |
| *Relevance items* | *12* | *Report if, and how, it was tested that all items are relevant for the construct of interest, for the target population of interest and for the context of use of interest.* |
| *Comprehensiveness items* | *13* | *Report if, and how it was tested that no key concepts are missing* |
| *Comprehensability items* | *14* | *Report if, and how was tested that instructions, items and reponse options are understood by the population of interest as intended.* |
| Results | | |
| Attrition | 15 | Indicate participant attrition throughout the study; report attrition rates both by monitoring days and waves, if applicable |
| Prompt delivery | 16 | Report number of EMA prompts that were planned to be delivered. If possible, also report the number of EMA prompts that were actually received by participants and indicate reasons for why prompts were not sent out (eg, technical issues or participant noncompliance reason such as phone was powered off) |
| Latency | 17 | Report the amount of time from prompt signal to answering of prompt |
| Compliance rate | 18 | Report total answered EMA prompts across all subjects and the average number of EMA prompts answered per person. Report compliance rate both by monitoring days and waves, if applicable. Indicate reasons for noncompliance, if known |
| Missing date | 19 | Report whether EMA compliance is related to demographic or time-varying variables |
| Discussion | | |
| Limitations | 20 | Discuss limitations of the study, taking into account sources of potential bias when using EMA methods (eg, reactivity, use of technology) |
| Conclusions | 21 | Provide a general interpretation of results and discuss the benefits of using EMA(eg, improving understanding of daily behaviours) |
